# Bone Marrow-Derived Mesenchymal Stem Cells and *γ*-Secretase Inhibitor Treatments Suppress Amyloid-*β*25–35-Induced Cognitive Impairment in Rat Dams and Cortical Degeneration in Offspring

**DOI:** 10.1155/2023/2690949

**Published:** 2023-05-25

**Authors:** Asmaa Gaber, Ahlam M. Elbakry, Rabab M. Aljarari, Fatima A. Jaber, Yasser A. Khadrawy, Dina Sabry, Rasha E. Abo-ELeneen, Osama M. Ahmed

**Affiliations:** ^1^Comparative Anatomy and Embryology Division, Department of Zoology, Faculty of Science, Beni-Suef University, P.O. Box 62521, Beni Suef, Egypt; ^2^Department of Biology, College of Science, University of Jeddah, Jeddah 21589, Saudi Arabia; ^3^Medical Physiology Department, Medical Branch Department, National Research Center, Giza, Egypt; ^4^Medical Biochemistry and Molecular Biology Department, Faculty of Medicine, Badr University in Cairo, Cairo 11829, Egypt; ^5^Medical Biochemistry and Molecular Biology Department, Faculty of Medicine, Cairo University, Cairo 11562, Egypt; ^6^Physiology Division, Department of Zoology, Faculty of Science, Beni-Suef University, P.O. Box 62521, Beni Suef, Egypt

## Abstract

Alzheimer's disease (AD) is the most frequent cause of age-related neurodegeneration and ensuing cognitive impairment. Progressive deposition of extracellular amyloid beta (A*β*) aggregates (plaques) and intracellular hyperphosphorylated Tau protein (p-Tau) are the core pathological markers of AD but may precede clinical symptoms by many years, presenting a therapeutic window of opportunity. Females are more frequently afflicted by AD than males, necessitating evaluation of novel treatments for the female population. The current study examined the protective efficacies of intravenous bone marrow-derived mesenchymal stem cells (BM-MSCs) and oral gamma-secretase inhibitor-953 (GSI-953) during pregnancy on cognitive impairment in rat dams and neurodegeneration in offspring induced by intracerebroventricular injection of A*β*25–35 prior to pregnancy. The A*β*25–35 (AD) group exhibited significant (*P* < 0.001) impairments in the Y-maze and novel object recognition test performance prior to conception. Histological analysis of the offspring cortex revealed substantial dendritic shrinkage and activation of microglial cells, while neurochemical analysis demonstrated significant increases in the proinflammatory cytokine interleukin-1*β* (IL-1*β*) and tumor necrosis factor-*α* (TNF-*α*). In contrast, BM-MSC or GSI-953 treatment of dams following A*β*25–35 injection significantly (*P* < 0.001) reduced the number and size of activated microglial cells, markedly increased dendrite length, and reversed proinflammatory cytokine elevations in offspring. Moreover, BM-MSC or GSI-953 treatment reversed the A*β*25–35-induced amyloid precursor protein and p-Tau elevations in the offspring brain; these changes were accompanied by upregulation of the brain-derived neurotrophic factor and downregulation of glycogen synthase kinase-3*β* in the serum and brain. Treatment with BM-MSCs or GSI-953 also reversed A*β*25–35-induced elevations in different gene expressions in the neonatal cortex. Finally, treatment of dams with BM-MSCs or GSI-953 prevented the A*β*25–35-induced disruption of newborn brain development. Thus, BM-MSC and GSI-953 treatments have broad-spectrum effects against A*β*25–35-induced brain pathology, including the suppression of neural inflammation, restoration of developmental plasticity, and promotion of neurotrophic signaling.

## 1. Introduction

Alzheimer's disease (AD) is an untreatable neurodegenerative condition that accounts for 60%–80% of global dementia cases [1]. Currently, about 50 million people worldwide suffer from AD, and epidemiological analyses predict that case numbers will reach 152 million by 2050, a roughly sixfold increase since 2006 [2]. Further, the prevalence of AD is higher in females than in males. Alzheimer's pathology is characterized by progressive neuronal cell death in a wide area of the cerebral cortex, basal forebrain, and hippocampus, causing cognitive impairments, such as memory loss, impaired decision-making, and language difficulties, in addition to emotional impairments [3–6]. The pathological hallmarks of AD include the extracellular deposition of amyloid proteins in plaques, the development of intracellular neurofibrillary tangles (NFTs) containing hyperphosphorylated- (p-) Tau protein, neuroinflammation, and various changes in the synaptic structure [6–8]. Many studies have proposed that AD pathogenesis involves multiple neurodegenerative processes triggered by plaque accumulation and that all of these pathogenic mechanisms are influenced by various AD risk genes [9]. Among the most important degenerative processes is the reactive oxygen species generation by amyloid plaques, which in turn causes oxidative cell damage and triggers inflammatory cascades [10].

Neurogenesis and synaptic plasticity, two long-lasting alterations in the mother's brain, are linked to important physiological changes throughout pregnancy [11]. During normal pregnancy, amyloid beta metabolism is upregulated; consequently, the impairment in production or clearance of amyloid beta is responsible for abnormal processes of the central nervous system. Placental dysfunction and amyloid beta peptides are related, according to Lederer et al. [12]. From the prenatal to early postnatal period, epigenomic dysregulation can have a deleterious impact on health. It has been established that exposure to various settings results in illnesses, epigenetic alterations, and abnormalities in neurodevelopment [13].

It is believed that AD's pathophysiological process begins before diagnosis [14]. At the preclinical stage of AD, significant cognitive impairment may already exist [15]. In the early stages of AD, episodic memory is initially damaged by a deficit in the limbic regions of the brain, which later spreads to the cortical regions of the brain; additional cognitive symptoms arise [16]; and the dementia syndrome is seen, according to imaging-based research [17, 18]. According to the hypothesis underlying the name “preclinical AD,” which was created to reflect this lengthy period of silence to support longitudinal clinical research investigations, preventive measures may be more effective in minimizing preclinical manifestation of AD dementia if applied early on [19]. Although familial AD is caused by genetic defects, the clinical manifestation of AD does not appear until middle life or later. Investigating whether beginning medication therapy while pregnant can act as a therapeutic preventative against AD pathogenesis is therefore a sensible idea [20].

Many drugs have been tested for AD treatment, but most target symptoms only, and none are able to reverse or stop disease progression. Further, only 20% of patients benefit from currently available AD drugs. Given the importance of amyloid plaque deposition and NFT formation in the early stages of AD, drug therapies that can reduce the amyloid peptide load, inhibit Tau phosphorylation, or protect vulnerable neurons against downstream pathogenic processes may prevent the associated cognitive dysfunction. Amyloid plaques are generated by the enzymatic cleavage of the transmembrane amyloid precursor protein (APP) by beta- and gamma-secretases [21]. Gamma-secretase is a multiple subunit complex that catalyzes the final step of amyloid peptide generation, so inhibition of this enzyme can prevent amyloid plaque aggregation [22, 23]. One such *γ*-secretase inhibitor-953 (GSI-953) is begacestat, which was found to reduce plasma amyloid peptide concentration in a phase I trial. Begacestat is 15 times more selective for the suppression of the APP cleavage as evidenced by cellular assays of Notch [24]. The same authors reported that high-dose GSI-953 substantially reduced cleaved amyloid beta 1–40 (A*β*1–40) levels in the brain, cerebrospinal fluid (CSF), and plasma, while low doses still reduced A*β*1–40 in the brain and plasma [24]. Importantly, it has been revealed that begacestat improves contextual memory impairments in Tg2576 transgenic mice [24]. Begacestat also improved contextual memory impairments in the Tg2576 transgenic mouse model of AD [24], suggesting promise as an AD treatment, although further study is required.

In addition to small-molecule drugs that inhibit AD-associated pathogenic processes, there is growing interest in stem cell therapy as a neuronal replacement and (or) protective strategy [25–27]. Indeed, stem cell therapy has shown promising results in animal models of neurodegenerative disease [28, 29]. Pluripotent stem cells (iPSCs), brain-derived neural stem cells, and bone marrow-derived mesenchymal stem cells (BM-MSCs) [30, 31] are the most widely used stem cell types in research on therapeutic applications as cells can differentiate into a wide variety of functional cells such as neural cells [32]. In comparison to conventional drug therapies, stem cell-based therapy may be more effective for AD because implanted stem cells can improve the brain microenvironment by supplying growth-promoting and growth-permissive factors for synaptogenesis and neurite repair, reduce oxidative stress by enhancing local antioxidant capacity, and trigger the sustained production of neurotrophic factors like brain-derived neurotrophic factor (BDNF) and nerve growth factor (NGF) [33].

Early AD treatment provides an opportunity to prevent or slow behavioral and synaptic dysfunction, pathogenic processes known to start long before clinical diagnosis [34]. Therefore, we evaluated the efficacies of MSCs and GSI-953 against A*β*25–35-induced cognitive dysfunction in adult females as well as against neurodegeneration in the offspring of these females.

## 2. Materials and Methods

### 2.1. Experimental Animals

Ninety Wistar rats, 60 mature females weighing 200–250 g and 30 mature males, were used in the current investigation. The animals were procured from the VACERA animal housing facility in Egypt. To prevent infections, animals were kept under surveillance at the Department of Zoology animal house in well-ventilated stainless-steel cages under a regular daily dark/light cycle, controlled humidity (50%), and controlled air temperature (23°C). Animals were fed a conventional rat pellet meal in addition to some vegetables as a source of vitamins and had unlimited access to tap water. The animal care protocol adhered to the Beni-Suef College Animal Care and Use Committee's general recommendations (approval number 019-75).

### 2.2. Surgical Procedure

Surgeries were conducted as described previously [35]. Free A*β*25–35 was dissolved in 0.9% saline to 1 mg/mL and incubated for 4 days at 37°C. Rats received intraperitoneal injections of 7 mg/kg xylazine and 70 mg/kg ketamine for deep anesthesia, after which head hair was removed using surgical shears. The shaved area was then sanitized with betadine and covered with a biodegradable surgical towel. An incision was made along the median longitudinal calvaria using surgical bistouries and scissors, and the subcutaneous tissue and fascia were gently separated. Sterile dry cotton was used to stop the bleeding, and the bregma was marked with a pen. A flexible bone drill was used to make a 1 mm diameter hole at 0.8 mm posterior (P) to the bregma and 2.0 mm lateral (L) to the midline (above the lateral ventricle). The bleeding was stopped and the skull surface cleaned repeatedly with sterile cotton. A needle linked to a Hamilton microsyringe was gently inserted through the bore hole to a depth of 4.6 mm and 10 *μ*L of A*β*25–35 slowly injected. The needle was left in place for 2 min to allow full emptying of the syringe contents into the lateral ventricle. Other rats were injected with equal-volume 0.9% saline as a control. Finally, the wound was sterilized with betadine, and the injured skin was closed using a simple suture method. One week after surgery, the rats were tested in the Y-maze and novel object recognition tests to evaluate working memory and reference memory, respectively. At the proestrus stage, the rats were mated with a male for one or two days. The first day of pregnancy was identified by checking a vaginal smear for sperm.

### 2.3. Animal Grouping (Figure 1)

Five groups of pregnant Wistar rats were established: G1 (*n* = 10) receiving intracerebroventricular (i.c.v.) injection of 10 *μ*L saline (0.9%) before pregnancy, G2 receiving i.c.v. A*β*25–35 before pregnancy (*n* = 30), G3 receiving intravenous (i.v.) Dulbecco's Modified Eagle Medium (DMEM) (4.5%), G4 group receiving i.v. injection BM-MSCs one time per week for three weeks during the pregnancy period starting ten days following i.c.v. injection of 10 *μ*L A*β*25–35, and G5 receiving 2.5 mg/kg·b·wt day after day oral begacestat during the pregnancy period starting ten days after 10 *μ*L i.c.v. A*β*25–35. The brains of the offspring were removed on postnatal days 7, 14, and 21 for histological, immunohistochemical, and neurochemical analyses.

### 2.4. Isolation of Bone Marrow Mesenchymal Stem Cells from Rats (Figure 2)

Mesenchymal stem cells were allogeneically isolated from the rat bone marrow as described in a previous study [36]. Briefly, the bone marrow was flushed out of the femur using 4.5% DMEM (Life Science Group Ltd., UK) and the suspension centrifuged at 3000 rpm for 5 min. Isolated BM cells were seeded in culture flasks with complete medium composed of 4.5% DMEM, 15% fetal bovine serum (Lonza Verviers Sprl, Belgium), and 1% penicillin-streptomycin (Life Science Group Ltd., UK) and grown in an atmosphere of 5% CO_2_ with 50% humidity at 37°C. The medium was exchanged after 3–4 days. When the cells reached 80%–90% confluence (7 to 10 days after seeding), they were washed twice with a phosphate-buffered saline (PBS), harvested via incubation in 0.25% trypsin/1 mM EDTA (Greiner Bio-One, Germany) for five minutes at 37°C, centrifuged, and resuspended in DMEM. Before injection, the viability was determined by combining 10 *μ*L of the cell suspension with 10 *μ*L of 0.4% trypan blue stain, and then, stained (dead/dying) cells were counted using a hemocytometer. Cells that demonstrated 95% viability were used for injection. Animals were injected with an estimated one million cells per week for 3 weeks. No side effect was detected during the study in response to MSC injection.

### 2.5. Behavioral Tests

#### 2.5.1. Y-Maze Test (as Spatial Memory Task)

The Y-maze task is used to gauge spatial working memory. The wooden Y-maze consists of three equally spaced arms (projecting 120° relative to adjacent arms), each 50 cm long, 5 cm wide at the hub, and 10 cm wide at the end, and is bounded by 20 cm high walls. Individual rats are placed on one arm and allowed to freely explore both that arm and an adjacent arm for 8 min, while the third arm is blocked by a partition. The rat is then allowed to freely explore all arms for 30 minutes. The number of entries, amount of time spent in the previously blocked (novel) arm, and amount of time spent in the two previously explored (familiar) arms were recorded. The proportion (%) of time spent in the novel arm relative to the familiar arms was calculated as an index of working memory [37].

#### 2.5.2. Novel Object Recognition Test

The novel object recognition test is a three-day protocol for evaluating long-term memory [38]. On the first day, all animals were acclimated to a 30 × 30 × 30 cm empty wooden cage for 10 min [38, 39]. On day two, animals were placed individually in the same cage now containing two identical wooden objects placed at opposite corners 2 cm from the walls and allowed to explore freely for 10 min [40].

On the third day, one of the objects was exchanged with another of different form, size, and color, and the rat was allowed five minutes to explore. The amount of time spent examining the novel object as a fraction of the total time spent exploring both objects was calculated as the recognition index (RI) [38]. A higher RI is considered a sign of better recognition memory [41].

### 2.6. Histological Analysis of Newborn Cortex

The whole cortex was isolated from 3 newborns per treatment group (G1–G5), fixed in 4% paraformaldehyde for 48 h (pH 7.4) at 4°C, dehydrated in gradient ethanol, and embedded in paraffin. Sections (5 *μ*m) were prepared and stained with hematoxylin and eosin (H&E) at room temperature for 12 min and photographed under light microscopy for evaluation of histopathological changes.

### 2.7. Immunohistochemistry

Newborns (four per treatment group) were anesthetized and perfused intracardially with 4% paraformaldehyde in 0.9% saline. Brain sections were cut at 5 *μ*m, blocked with 1% bovine serum albumin (BSA) in PBS containing 0.3% Triton X-100 for 1 h, and incubated with an antibody against the microglial marker-ionized calcium-binding adapter molecule 1 (Iba-1) (1 : 1000, Abcam, Cambridge, UK) overnight at 4°C. Sections were then incubated with a corresponding secondary antibody (1 : 1000) for 1 h, and immunostaining was visualized using an Olympus microscope equipped with a 10x and 100x objective lens. Images were analyzed using ImageJ (NIH, Bethesda. MD, USA).

### 2.8. ELISA Assay

Alterations in inflammatory cytokines (IL-1*β* and TNF*α*), GSK-3*β*, and BDNF in the newborn serum were detected using ELISA kits of IL-1*β* (SEA563Ra) (Cloud-Clone Corp.), TNF-*α* (BioLegend), GSK-3*β* (Abbexa LLC, Houston, TX, USA), and BDNF (SEA011Ra) (Cloud-Clone Corp.). All the ELISA kits were used in accordance with the manufacturer's instructions (*n* = 6 per group); the quantity of these factors was expressed as pg/mL.

### 2.9. RT-PCR

Total RNA was isolated from the cortex lysate using the Direct-zol RNA Miniprep Plus kit (cat.# R2072, Zymo Research, Irvine, CA, USA) according to the manufacturer's instructions and reverse transcribed using the Superscript IV One-Step RT-PCR kit (cat.# 12594100, Thermo Fisher Scientific, Waltham, MA, USA) in accordance with the manufacturer's instructions. The product cDNA was amplified using 2x Platinum™ SuperFi™ RT-PCR Master Mix and primer pairs for the target genes (BDNF, NF-*κβ*, TNFR, TGF-*β*, and caspase-3) listed in Table 1. Quantitative real-time PCR (qRT-PCR) was conducted using SYBER green (StepOne, Applied Biosystem, Foster City, USA), and expression levels of target genes were calculated as the cycle threshold (Ct) relative to that of the *β*-actin gene using the delta-delta Ct (Ct) method.

### 2.10. Western Blotting

To assess brain concentrations of p-Tau and APP, the total protein was extracted from cerebral cortex samples using a total protein extraction kit (Bio-Rad cat.# 163-2086) in accordance with the manufacturer's instructions and measured via a colorimetric assay (Bio-Rad, Hercules, CA, USA, cat.# 163-2086). Equal amounts of protein were electrophoretically separated via sodium dodecyl sulfate polyacrylamide gel electrophoresis and transferred to polyvinylidene fluoride (PVDF) membranes. Membranes were incubated in a stopping buffer (tris-buffered saline with Tween 20 (TBST)) containing 3% BSA for 1 hour at room temperature and then in primary antibodies against phospho-Tau (Thr231), APP, and *β*-actin (as the gel-loading control) overnight at room temperature. The blot was then washed 3–5 times in TBST and immunoreactive bands visualized using chemiluminescent substrate (Clarity™ Western ECL substrate, Bio-Rad cat.# 170-5060). Target band signals were then identified using a CCD camera-based imager (ChemiDoc MP imager) and quantified relative to *β*-actin signals using the accompanying image analysis software.

### 2.11. Statistical Analysis

All statistical analyses were conducted using SPSS version 17.0 (SPSS Inc., 1989–2007; Chicago, IL, USA). Data are presented as mean ± standard error of the mean after one-way ANOVA analysis. The effects of age, therapy, and their interactions on most outcomes were examined via two-way ANOVA followed by Tukey's multiple comparison tests. Behavioral test results were evaluated via one-sample *T*-test. The significance was considered at three levels, *P* < 0.05, *P* < 0.01, and *P* < 0.001, while *P* > 0.05 was nonsignificant.

## 3. Results

### 3.1. Intracerebroventricular A*β*25–35 Injection Impaired Working Memory and Object Recognition Memory in Adult Female Rats

**Figure 3:**
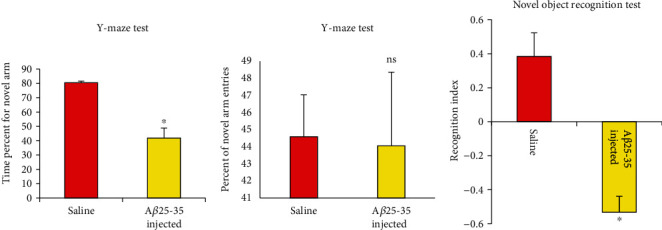
Effect of A*β*25–35 injection into adult female rats before pregnancy on behavioral test. (a, b) Y-maze test; (c) novel object recognition test. Data are presented as mean ± SEM. ^∗^*P* < 0.001. ns: insignificant for the percent of novel arm entries comparing with the saline-injected group (*P* > 0.05).

The Y-maze test (Figures 3(a) and 3(b)) revealed significant working memory impairment (*P* < 0.001) among rats injected i.c.v. with A*β*25–35 (group G2) as evidenced by the reduced time spent in the novel arm compared to rats receiving i.c.v. saline (group G1). In contrast, there were no group differences in the number of novel arm entries, suggesting no significant effects of treatment on motor activity or exploratory drive (*P* > 0.05). In addition, G2 rats demonstrated a significant (*P* < 0.001) reference memory impairment compared to G1 rats as evidenced by the reduced novel object exploration during the test phase of the novel object recognition task (Figure 3(c)).

### 3.2. Intracerebroventricular A*β*25–35 Injection in Dam Disrupted Cortical Development in Offspring, an Effect Reversed by BM-MSC and GSI-953 Treatments

**Figure 4:**
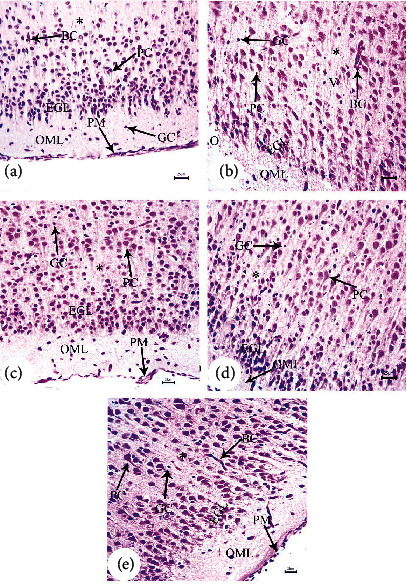
Photomicrograph of hematoxylin-and-eosin- (H&E-) stained sections of the neonatal cerebral cortex at PND7 of the following groups: (a) saline-injected group that illustrates that the normal organization of cells of the prefrontal cortex displays the external layers of the cortex that have the outer molecular layer (OML) underneath the regular attached pia mater (PM); both the external granular (EGL) and pyramidal layers exhibit small neuronal cell bodies having rounded open face nuclei (PC) that are surrounded with a little cytoplasm, normal capillaries (BC), and normal glial cells (GC) that are seen within the acidophilic neuropil (^∗^). (b) A*β*25–35-injected group demonstrates disrupted and fragmented attached pia mater (PM) and disorganization of cells of the prefrontal cortex with decreased external granular layer thickness (EGL), degenerated neuronal cells (dN) with pyknotic nuclei and elongated filament, marked dilated congested inflamed capillaries (BC), neuropil (^∗^) with marked edema (O), and degenerated glial cells (GC). (c) DMEM group displayed normal cerebral cortex architecture. (d, e) AD treated with MSCs and AD treated with GSI-953 showed normally attached pia mater and normally arranged cells of the prefrontal cortex having normal cellular thickness layer, completely regenerated neuronal cells (PC), normal capillaries (BC), absence of edema, and completely regenerated glial cells (GC) (scale bar = 25 *μ*m).

**Figure 5:**
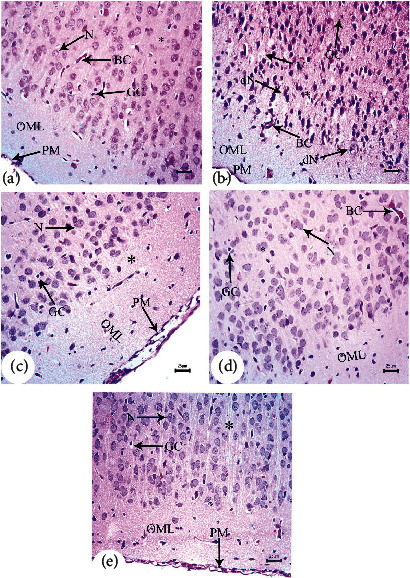
Photomicrograph of hematoxylin-and-eosin (H&E-) stained sections of the neonatal cerebral cortex at PND14 of the following groups: (a) saline-injected group that illustrates that the normal organization of cells of the prefrontal cortex displays the external layers of the cortex that have the outer molecular layer (OML) underneath the regular attached pia mater (PM); both the external granular (EGL) and pyramidal layers exhibit small neuronal cell bodies having rounded open face nuclei (N) that are surrounded with a little cytoplasm, normal capillaries (BC), and normal glial cells (GC) that are seen within the acidophilic neuropil (^∗^). (b) A*β*25–35-injected group demonstrates disrupted and fragmented attached pia mater (PM) and disorganization of cells of the prefrontal cortex with decreased external granular layer thickness (EGL), degenerated neuronal cells (dN) with pyknotic nuclei, and elongated filament and pericellular haloes (PV); some of these cells had pointed ends. There were many glial cell nuclei in a vacuolated neuropil and dilated, crowded blood capillaries. Rarely visible were typical pyramidal cell bodies, marked dilated congested inflamed capillaries (BC), neuropil (^∗^) with marked vacuoles (V), and degenerated glial cells (GC). (c) DMEM group displayed a normal histological structure of cerebral cortex. (d, e) AD treated with MSCs and AD treated with GSI-953 showed normally attached pia mater and normally arranged cells of the prefrontal cortex having a normal cellular thickness layer, completely regenerated neuronal cells (N), normal capillaries (BC), absence of edema, and completely regenerated glial cells (GC) (scale bar = 25 *μ*m).

**Figure 6:**
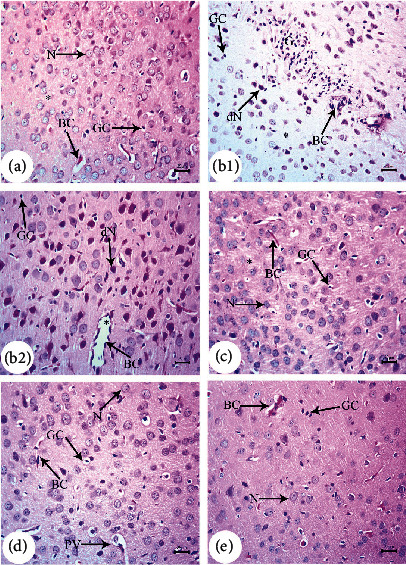
Photomicrograph of hematoxylin-and-eosin- (H&E-) stained sections of the neonatal cerebral cortex at PND21 of the following groups: (a) saline-injected group that illustrates that the normal pyramidal layers exhibit small neuronal cell bodies that have rounded open face nuclei (N) that are surrounded with a little cytoplasm, normal capillaries (BC), and normal glial cells (GC) that are seen within the acidophilic neuropil (^∗^). (b1, b2) A*β*25–35-injected group demonstrates degenerated neuronal cells (dN) with pyknotic nuclei and elongated filament and pericellular haloes (PV); some of these cells had pointed ends. There were many glial cell nuclei with marked gliosis (G) in a vacuolated neuropil and dilated, crowded blood capillaries. Rarely visible were typical pyramidal cell bodies, marked dilated congested inflamed capillaries (BC), neuropil (^∗^) with marked vacuoles (V), and degenerated glial cells (GC). (c) DMEM group displayed normal cerebral cortex architecture. (d, e) AD treated with MSCs and AD treated with GSI-953 showed completely regenerated neuronal cells (N), normal capillaries (BC), absence of edema, and completely regenerated glial cells (GC) (scale bar = 25 *μ*m).

H&E-stained parasagittal slices of the prefrontal cortex (PFC) from the PND7 offspring of saline-injected (G1) dams (Figure 4(a)) exhibited an intact pia mater layer, regular arrangements of neurons in each cortical layer, and normal cellular structures. The pia mater layer was acidophilic and contained abundant blood capillaries as well as neuronal and glial cell processes. The neuronal cell bodies in inner cortical layers exhibited large nucleoli and rounded open face nuclei encircled by the basophilic cytoplasm. Inner-layer neurons were interspersed with neuroglial cell nuclei. Pyramidal cells in the cerebral cortex changed in size and shape from PND7 to PND21, consistent with the normal cortical development (Figures 5(a) and 6(a)).

**Figure 7:**
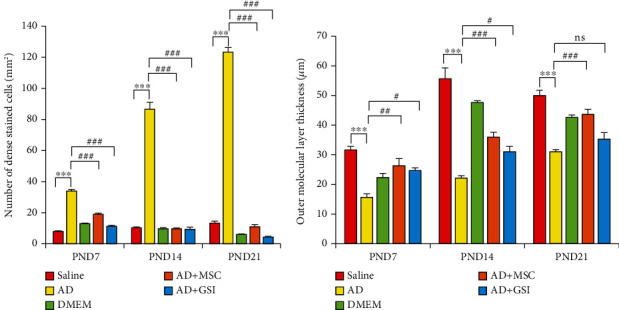
Effect of treatment with MSCs and GSI-953 on (a) number of degenerated neural cells/mm, (b) outer molecular layer thickness (*μ*m) in the newborn cortex of A*β*25–35-induced Alzheimer's disease dams. Data were analyzed via two-way ANOVA followed by Tukey's multiple comparison test. Data are expressed as mean ± SEM. ^∗^*P* < 0.05, ^∗∗^*P* < 0.01, and ^∗∗∗^*P* < 0.001 vs. the saline-injected group. ^#^*P* < 0.05, ^##^*P* < 0.01, and ^###^*P* < 0.001 vs. the AD group. ^ns^*P* > 0.05 no significant.

In contrast, the arrangement of cells in cortical slices from G2 offspring at PND7 (Figure 4(b)) was disturbed, and the external granular layer thickness was significantly (*P* < 0.001) reduced throughout all tested periods (Figure 7(b)). Neuronal cells (dN) of the granular layer exhibited pyknotic nuclei and elongated filaments interspersed with dilated and congested blood capillaries (BC), regions of marked edema (O), and degraded glial cells (GC). Similarly, parasagittal slices of the PND14 and PND21 prefrontal cortex showed evident disturbances in the cortical layer arrangement with dilated BC (Figures 5(b), 6(b1), and 6(b2)). The PFC of offspring from G2 dams also exhibited darkly stained and shrunken neuronal cell bodies with intensely stained pyknotic nuclei (dN) and pericellular vacuoles (PV) (Figures 5(b), 6(b1), and 6(b2)). Some of these cells had pointed ends. The neuropil was also dilated and vacuolated with many glial cell nuclei interspersed among closely spaced, dilated capillaries (Figures 6(b1) and 6(b2)). Moreover, few typical pyramidal cell bodies were visible. From PND7 to PND21, the cerebral cortex exhibited a significant (*P* < 0.001) rise in degenerating neurons, and pronounced gliosis was observed at PND21 (Figures 6(b1) and 7(a)). This A*β*-induced degeneration was markedly reversed via BM-MSC and GSI-963 treatments as shown in Figures 4(d), 4(e), 5(d), 5(e), 6(d), and 6(e), as slices from the G4 and G5 offspring exhibited a fully intact pia mater layer and normal cellular organization within cortical layers as well as regenerated neuronal cells (N), mildly dilated and inflamed congested capillaries (BC), mild edema (O), and regenerated glial cells at PND7–PND21. The offspring's cortexes from the DMEM group showed a normal histological structure relative to those from the saline-injected group (Figures 4(c), 5(c), and 6(c)).

### 3.3. BM-MSC and GSI-953 Treatments Restored near Normal Microglial Cell Counts, Soma Size, and Processes Length in the Cortex of Offspring from A*β*25–35-Injected Dams

**Figure 8:**
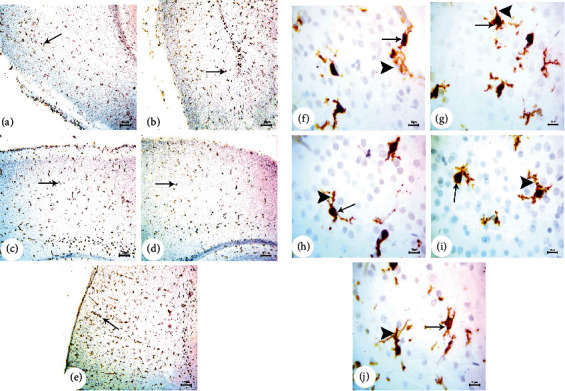
Photomicrographs (a) of immunohistochemical study for neonatal cortex using Iba-1 to illustrate the microglial cell reactivity at PND7: (a) saline-injected group, (b) A*β*25–35 group, (c) DMEM group, (d) AD+MSC group, and (e) AD+GSI. Scale bar 100 *μ*m (10x). Photomicrographs (b) of immunohistochemical study for the neonatal cortex using Iba-1 to illustrate the microglial cell reactivity at PND7: (f) saline-injected group, (g) A*β*25–35 group, (h) DMEM group, (i) AD+MSC group, and (j) AD+GSI. Scale bar 10 *μ*m (100x). Arrows refer to the cell soma size; arrowheads refer to the length, thickness, and number of dendrites.

**Figure 9:**
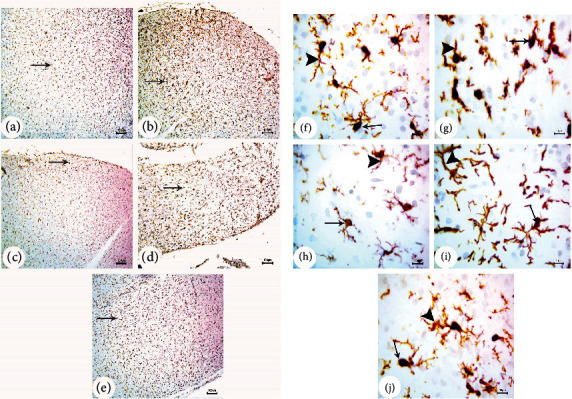
Photomicrographs (a) of the immunohistochemistry study for the neonatal cortex using Iba-1 to illustrate the microglial cell reactivity at PND14: (a) saline-injected group, (b) A*β*25–35 group, (c) DMEM group, (d) AD+MSC group, and (e) AD+GSI. Scale bar 100 *μ*m (10x). Photomicrographs (b) of the immunohistochemistry study for the neonatal cortex using Iba-1 to illustrate the microglial cell reactivity at PND14: (f) saline-injected group, (g) A*β*25–35 group, (h) DMEM group, (i) AD+MSC group, and (j) AD+GSI. Scale bar 10 *μ*m (100x). Arrows refer to the cell soma size; arrowheads refer to the length, thickness, and number of dendrites.

**Figure 10:**
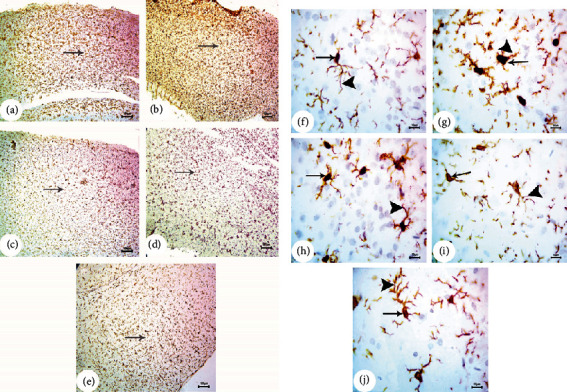
Photomicrographs (a) of the immunohistochemistry study for the neonatal cortex using Iba-1 to illustrate the microglial cell reactivity at PND21: (a) saline-injected group, (b) A*β*25–35 group, (c) DMEM group, (d) AD+MSC group, and (e) AD+GSI. Scale bar 100 *μ*m (10x). Photomicrographs (b) of the immunohistochemistry study for the neonatal cortex using Iba-1 to illustrate the microglial cell reactivity at PND21: (f) saline-injected group, (g) A*β*25–35 group, (h) DMEM group, (i) AD+MSC group, and (j) AD+GSI. Scale bar 10 *μ*m (100x). Arrows refer to the cell soma size; arrowheads refer to the length, thickness, and number of dendrites.

**Figure 11:**
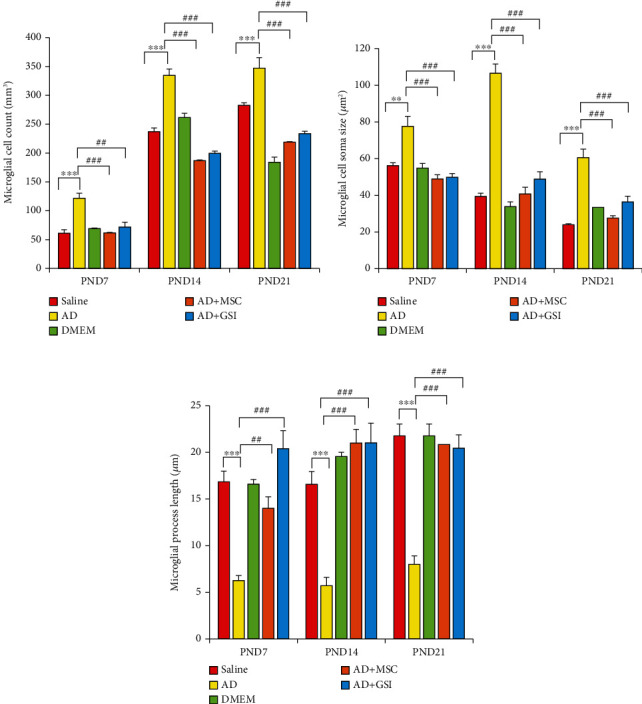
Effect of treatment with MSCs and GSI-953 on (a) microglial cell count/mm^3^, (b) cell soma size, (c) processes length in the newborn cortex of A*β*25–35-induced Alzheimer's disease dams. Data were analyzed via two-way ANOVA followed by Tukey's multiple comparison test. Data are expressed as mean ± SEM. ^∗^*P* < 0.05, ^∗∗^*P* < 0.01, and ^∗∗∗^*P* < 0.001 vs. the saline-injected group. ^#^*P* < 0.05, ^##^*P* < 0.01, and ^###^*P* < 0.001 vs. the AD group. ns: nonsignificant (*P* > 0.05).

Amyloid-*β* deposition causes excessive activation of microglial cells and significantly accelerates AD development. Therefore, we quantified activated microglial cell numbers in the neonatal cortex via Iba-1 immunostaining (Figures 8, 9, and 10). In the A*β*25–35-injected group (G2), the number of Iba-1-positive cells was significantly enhanced, microglial soma size significantly greater, and processes length significantly reduced (all *P* < 0.001) from PND7 to PND21 compared to age-matched neonates of saline-injected dams (G1) (Figures 11(a)–11(c)). Consistent with qualitative histological analyses, BM-MSC and GSI-953 treatments significantly (*P* < 0.001) restored microglial number and soma size as well as processes length at PND7, PND14, and PND21. In addition to these main effects, two-way ANOVA showed a significant (*P* < 0.001) interaction between the treatment group and neonatal age, suggesting restoration of the normal cortical development.

Also, there was a significant main effect of the treatment group (*F*_4,75_ = 652.437, *P* < 0.001) and neonatal age (*F*_2,75_ = 4.758, *P* < 0.05) on serum TNF-*α* as well as a significant treatment group × age interaction (*F*_8,75_ = 78.646, *P* < 0.001).

### 3.4. BM-MSC and GSI-953 Treatments Restored Normal Serum Proinflammatory, Prodegenerative, and Neuroprotective Factor Concentrations in Offspring of A*β*25–35-Injected Dams

#### 3.4.1. Effects on Serum Neuroinflammatory Cytokine Concentrations

**Figure 12:**
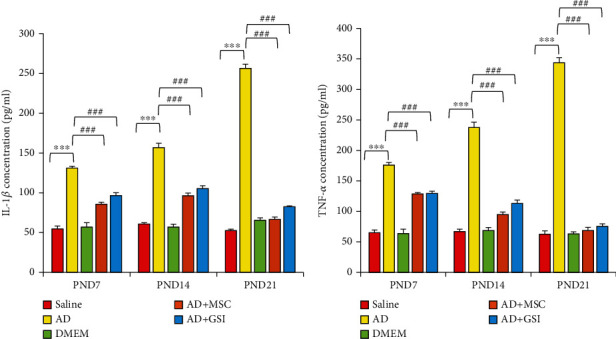
Effect of treatment with MSCs and GSI-953 on the neuroinflammatory cytokine concentration (pg/mg). (a) IL-1*β* and (b) TNF-*α* in the newborn cerebral cortex of A*β*25–35-induced Alzheimer's disease dams. Data were analyzed via two-way ANOVA followed by Tukey's multiple comparison test. Data are expressed as mean ± SEM. ^∗^*P* < 0.05, ^∗∗^*P* < 0.01, and ^∗∗∗^*P* < 0.001 vs. the saline-injected group. ^#^*P* < 0.05, ^##^*P* < 0.01, and ^###^*P* < 0.001 vs. AD group. ns: nonsignificant (*P* > 0.05).

To evaluate the efficacies of BM-MSCs and GSI-953 against A*β*25–35-induced neuroinflammation, we measure serum IL-1*β* and TNF-*α* in G2 newborns at different PNDs. Both IL-1*β* and TNF-*α* concentrations were significantly (*P* < 0.001) elevated in the G2 group relative to the saline-injected group (G1) at all PNDs examined (Figures 12(a) and 12(b)), while BM-MSC and GSI-953 treatment of dams significantly (*P* < 0.001) diminished both serum IL-1*β* and TNF-*α* levels at all tested PNDs. Specifically, the concentrations of IL-1*β* and TNF-*α* were significantly elevated in G2 offspring (*P* < 0.001) from PND7 to PND21. Two-way ANOVA revealed a significant main effect of the treatment on serum IL-1*β* among groups (*F*_4,75_ = 624.956, *P* < 0.001) and PNDs (*F*_2,75_ = 39.079, *P* < 0.001) as well as a significant group × age interaction (*F*_8,75_ = 86.077, *P* < 0.001).

#### 3.4.2. Effects on Serum GSK-3*β* and BDNF Concentrations

**Figure 13:**
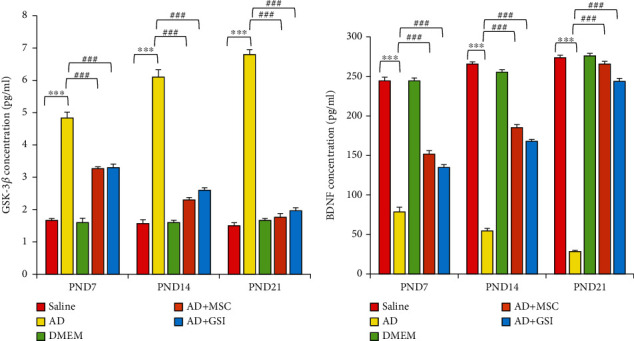
Effect of treatment with MSCs and GSI-953 on (a) GSK-3*β* and (b) BDNF concentration in newborn sera of A*β*25–35-induced Alzheimer's disease dams. Data were analyzed via two-way ANOVA followed by Tukey's multiple comparison test. Data are expressed as mean ± SEM. ^∗^*P* < 0.05, ^∗∗^*P* < 0.01, and ^∗∗∗^*P* < 0.001 vs. the saline-injected group. ^#^*P* < 0.05, ^##^*P* < 0.01, and ^###^*P* < 0.001 vs. the AD group. ns: nonsignificant (*P* > 0.05).

To assess the neuroprotective efficacies of MSCs and GSI-953 against i.c.v. A*β*25–35-induced neurodegeneration and Tau hyperphosphorylation, we measured the serum GSK-3*β* level and BDNF concentration in newborns (Figures 13(a) and 13(b)). Serum GSK-3*β* was significantly elevated while the BDNF concentration was significantly reduced in the serum of G2 newborns compared to G1 newborns at all tested PNDs (all *P* < 0.001), while both BM-MSC and GSI treatments significantly reversed these changes at all postnatal ages (all *P* < 0.001 vs. G2). Further, the serum GSK-3*β* level was significantly greater and the BDNF concentration significantly lower (both *P* < 0.001) at PND21 compared to younger ages, indicating a progressive degenerative process, while the two-way ANOVA revealed a significant main effect of the treatment group on serum GSK-3*β* (*F*_4,75_ = 699.489, *P* < 0.001), a significant main effect of the postnatal age on serum GSK-3*β* (*F*_2,75_ = 3.429, *P* < 0.05), and a significant group × time interaction (*F*_8,75_ = 36.289, *P* < 0.001). Similarly, there was a significant main effect of the treatment group on serum BDNF (*F*_4,75_ = 1594.494, *P* < 0.001), a significant main effect of the postnatal age on serum BDNF (*F*_2,75_ = 212.523, *P* < 0.001), and a significant group × time interaction (*F*_8,75_ = 89.763, *P* < 0.001).

### 3.5. BM-MSC and GSI-953 Treatments Restore Normal Expression Levels of Neuroprotective and Proapoptotic Genes in the Cortex of Offspring from A*β*25–35-Injected Dams

#### 3.5.1. Effects on BDNF, Caspase-3, TGF-*β*, NF-*κ*B, and TNFR Gene Expression in the Neonatal Cortex

**Figure 14:**
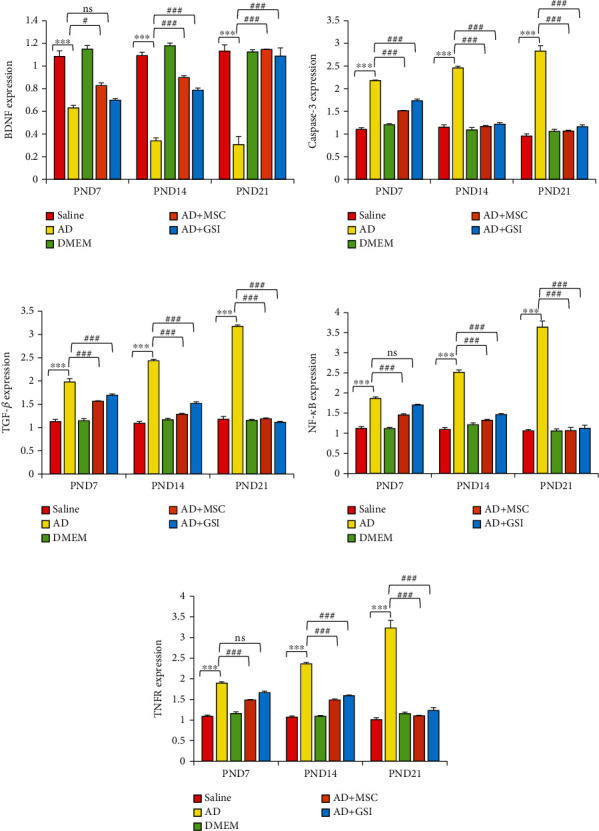
Effect of treatment with MSCs and GSI-953 on the gene expression of (a) BDNF, (b) cleaved caspase-3, (c) TGF-*β*, (d) NF-*κ*B, and (e) TNFR in the newborn cortex of A*β*25–35-induced Alzheimer's disease dams evaluated via qRT-PCR. Data were analyzed via two-way ANOVA followed by Tukey's multiple comparison test. Data are expressed as mean ± SEM. ^∗^*P* < 0.05, ^∗∗^*P* < 0.01, and ^∗∗∗^*P* < 0.001 vs. the saline-injected group. ^#^*P* < 0.05, ^##^*P* < 0.01, and ^###^*P* < 0.001 vs. the AD group. ns: nonsignificant (*P* > 0.05).

Expression levels of BDNF, caspase-3, TGF-*β*, NF-*κ*B, and TNFR genes were measured in the cerebral cortex of rat offspring via qRT-PCR (Figures 14(a)–14(e)). The expression level of BDNF mRNA was significantly (*P* < 0.001) reduced in G2 offspring at all tested ages compared to G1 offspring, consistent with serum protein measurements, while BM-MSC and GSI-953 treatments significantly reversed this effect (*P* < 0.001). Further, the two-way ANOVA revealed a significant (*P* < 0.001) main effect of the treatment group, a significant main effect of the neonatal age, and a significant treatment group × age interaction. Further, mRNA expression levels of caspase-3, TGF-*β*, NF-*κ*B, and TNFR were significantly (*P* < 0.001) elevated in the G2 neonatal cortex compared to the G1 neonatal cortex.

Offspring of A*β*25–35-injected dams that received BM-MSC or GSI-953 treatment showed significant (*P* < 0.001) downregulation of caspase-3, TGF-*β*, NF-*κ*B, and TNFR genes relative to neonates of A*β*25–35-injected dams at all postnatal ages. Expression levels of caspase-3 (*P* < 0.001), TGF-*β* (*P* < 0.01), NF-*κΒ* (*P* < 0.01), and TNFR (*P* < 0.05) increased with the neonatal age, and there was a significant (*P* < 0.001) main effect of the group at all tested ages according to the two-way ANOVA.

#### 3.5.2. Effects on p-Tau and APP Protein Concentration in the Neonatal Cortex

Phosphorylated- (p-) Tau is a core pathological hallmark of AD and contributes to disease progression. We detected a significant elevation in the cortical p-Tau protein among the newborns of G2 dams compared to the offspring of G1 dams, while BM-MSC and GSI-953 treatments significantly (*P* < 0.001) reduced p-Tau in the cerebral cortex compared to the offspring of G2 dams at all ages examined.

**Figure 15:**
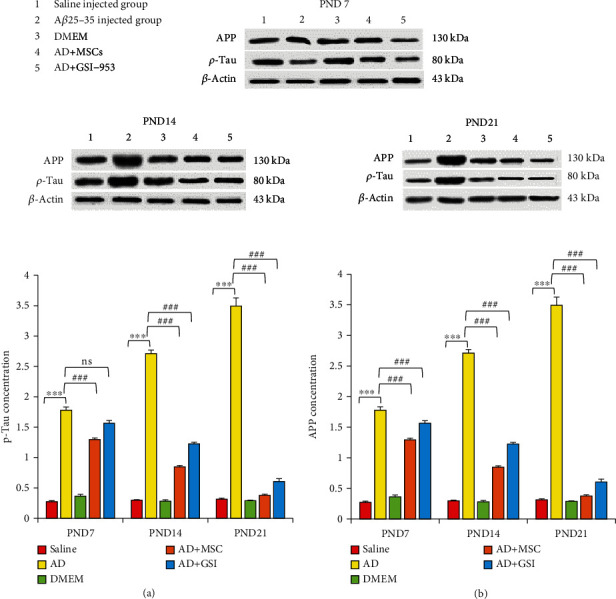
Effect of the treatment with MSCs and GSI-953 on (a) p-Tau and (b) APP protein concentration in the newborn cortex of A*β*25–35-induced Alzheimer's disease dams using the Western blotting technique. Data are expressed as mean ± SEM. ^∗^*P* < 0.05, ^∗∗^*P* < 0.01, and ^∗∗∗^*P* < 0.001 vs. the saline-injected group. ^#^*P* < 0.05, ^##^*P* < 0.01, and ^###^*P* < 0.001 vs. the AD group. ns: nonsignificant (*P* > 0.05).

The two-way ANOVA showed a significant main effect of the treatment group (*P* < 0.001) and group × age interaction (*P* < 0.001). Similarly, the APP protein concentration was significantly (*P* < 0.001) elevated in the cortex of the G2 offspring compared to the G1 offspring, while MSC and GSI-953 treatments significantly reversed (*P* < 0.001) this elevation at all neonatal ages examined (Figure 15). The two-way ANOVA also revealed a significant main effect of the treatment group and a group × age interaction (both *P* < 0.001).

## 4. Discussion

The prevalence of AD is expected to rise substantially due to gains in human longevity, which could place an insurmountable burden on healthcare systems [42] unless effective treatments are developed [43]. The main challenge for effective AD treatment is that the pathological processes begin long before the emergence of clinical symptoms, by which time much of the disease-associated damage may be irreversible. Therefore, the development of safe but effective preventative treatments may be the best strategy to reduce the disease burden. For this purpose, investigation of treatments effective against A*β* toxicity in the young adult and developing brain may be a fruitful approach [44].

Formation of the rat neural tube is completed by gestational day E10.5–11, while the mouse neural tube is completely formed by E9.5–10 [45]. Moreover, the cerebral cortex reaches adult weight by postnatal day 20. This rapid development may be particularly advantageous for the study of preventative AD treatments, and indeed, we demonstrate that BM-MSC injection or oral GSI-953 can protect against brain maldevelopment from the maternal A*β*25–35 injection. To elucidate the protective mechanisms of BM-MSC and GSI-953, we examined effects on neuroinflammatory signaling, microglial activation, cortical cell migration, neuronal development, neurotrophic factor signaling, and apoptosis. Several research papers demonstrated that Alzheimer's disease during pregnancy causes cognitive impairment in the neonates, and possible treatment starting early from prenatal to early postnatal periods may prevent cognitive deficits, reduce Tau and amyloid pathologies, and decrease postsynaptic deficits and neuroinflammation in the brain at the adult stage in 3xTg-AD mice [44].

**Figure 16:**
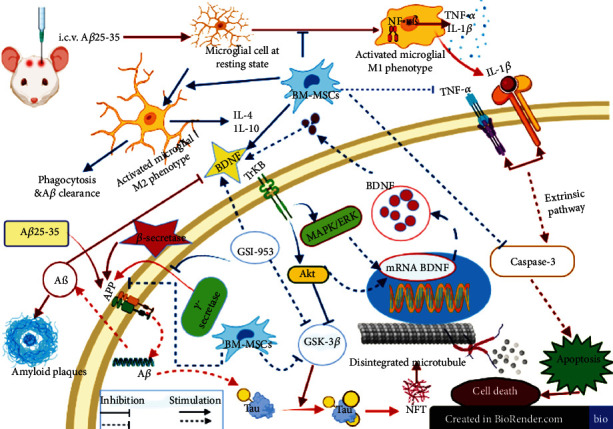
Pathways of MSCs and GSI-953 against the A*β*25–35-induced Alzheimer's disease in neonates. The figure was created with BioRender.com.

In the current investigation, we elucidated potential underlying mechanisms of action for BM-MSCs and GSI-953 as treatments for Alzheimer's disease brought on by a single intracerebroventricular injection of the A*β*25–35 rat model (Figure 16).

The first clinical manifestation of AD is memory impairment [46]. In animal models, i.c.v. A*β*25–35 injection is one of the most popular methods for induction of AD-like pathology as A*β*25–35 is converted into a fibril sheet that mimics the toxicity of A*β*1–40 [46, 47]. In the present study, the single i.c.v. injection of A*β*25–35 into adult female rats caused significant impairments in working memory and long-term memory as evidenced by poor Y-maze and novel object recognition test performance, respectively, consistent with previous reports [48, 49]. In addition, A*β*25–35 injection induced remarkable histopathological changes in the neonatal cerebral cortex, including distortion and disorganization of cortical layers, thinning of the external granular layer, and dilatation of blood vessels. These pathological signs progressed in severity from PND7 to PND21, consistent with a previous study demonstrating thinning of the cortex in patients with mild cognitive impairment, which is considered a precursor to clinical AD [50]. These histopathological changes were reversed via maternal BM-MSC and GSI-953 treatments. The protective effect of MSCs may be explained by metabolic compensation for dysfunctional mitochondria [51, 52], while GSI-953 may suppress the plaque burden, thereby reducing downstream inflammation and other harmful processes.

In addition to the accumulation of amyloid plaques and NFTs, AD pathology involves synaptic failure, oxidative stress, neuroinflammation, and mitochondrial dysfunction [42, 53]. Microglial cells are the resident macrophages of the central nervous system [42], and the pathological insult induces microglial cell activation, a state that triggers local inflammatory signaling and the recruitment of exogenous inflammatory cells [54]. Moreover, A*β*-induced microglial activation and downstream inflammatory responses further accelerate A*β* deposition [55], resulting in a self-sustaining neurogenerative condition. Injection of A*β*25–35 into the dam lateral ventricle significantly increased the number of activated microglial cells in the neonatal cortex, consistent with previous reports [47, 48], and this response was reversed by BM-MSC injection, again in accordance with previous findings [1, 56]. Panchenko et al. [57] found that intravenously injected MSCs pass through the blood-brain barrier and reach the temporal cortex and hippocampal dentate gyrus, possibly promoted by the phagocytic activity of activated microglial cells around A*β* deposits [58, 59]. In addition, we show for the first time that oral GSI-953 administration can also quell microglial cell activation in response to i.c.v. A*β*25–35 injection, possibly by reducing APP to A*β* conversion [24] through inhibition of *γ*-secretase activity.

The A*β* peptide is generated from APP through cleavage via *β*-secretase and *γ*-secretase [21]. The APP concentration was significantly elevated in the neonatal cortex of A*β*25–35-injected dams relative to saline-injected dams, while maternal BM-MSC and GSI-953 treatments during pregnancy reversed this rise. These findings are in agreement with Martone et al. [24], who reported that GSI-953 inhibits cleavage of APP and reduces A*β* production both *in vitro* and in a transgenic mouse model.

Tau is a microtubule-associated protein responsible for supporting the neuronal structure and synaptic function [60]. Amyloid-*β* deposition causes hyperphosphorylation of Tau kinases by activating GSK-3*β* [61, 62]. Injection of A*β*25–35 upregulated the expression of GSK-3*β* and increased the p-Tau concentration in the neonatal cortex, consistent with previous studies reporting a sustained rise in the p-Tau concentration within the frontal cortex following A*β*25–35 injection [48, 63]. Hyperphosphorylation reduces the affinity of Tau for microtubules, resulting in a reduced axonal transport and synaptic dysfunction [64, 65]. Maternal treatment with BM-MSCs or GSI-953 reversed this increase in p-Tau concentration, possibly by reducing GSK-3*β* activity [61, 66].

BDNF promotes neuronal differentiation and survival by suppressing proapoptotic proteins and upregulating neurofunctional proteins through the cyclic adenosine monophosphate response element binding protein transcription pathway [67, 68]. BDNF passes through the placenta *in vivo* and is also delivered via breast milk [44, 69]. Maternal A*β*25–35 injection significantly decreased the BDNF gene expression in the neonatal cortex (Figure 16) and reduced the serum concentration of the BDNF protein compared to offspring of saline-injected dams. Similarly, pro-BDNF, mature BDNF, and the corresponding mRNAs were reduced in the parietal cortex and hippocampus via A*β*25–35 injection [44]. On the contrary, maternal treatment with BM-MSCs and GSI-953 reversed this BDNF downregulation, possibly due to compensatory release from transplanted BM-MSCs [70] and (or) by the GSI-953-induced suppression of the GSK-3*β* activity.

Amyloid-*β* deposition stimulates the inflammatory response by increasing the cortical levels of TNF-*α*, IL-1*β*, and NF-*κ*B [71], which in turn increase the expression of APP and upregulate *γ*-secretase activity, causing a further rise in A*β* levels [72] (Figure 16). Activated microglial cells release the proinflammatory cytokines TNF-*α* and IL-1*β* in response to A*β*25–35 injection. Inactive NF-*κ*B is retained in the cytoplasm by binding to inhibitory *κβ* (I*κβ*). In response to various forms of stimulation, particularly those associated with stress, I*κβ* is phosphorylated and degraded, allowing NF-*κ*Bp^65^ migration into the nucleus where it enhances the expression of proinflammatory cytokines [73]. Maternal treatment with BM-MSCs or GSI-953 reduced the upregulation of proinflammatory signals induced by A*β*25–35 injection, possibly through inhibition of NF-*κβ* signaling [61]. In accordance with this notion, MSC transplantation into AD model mice suppressed the release of proinflammatory cytokines, such as TNF-*α*, IL-1*β*, and IL-6, from activated microglia and astrocytes [44, 59] and shifted microglial polarization from the proinflammatory M1 phenotype to the immunomodulatory M2 phenotype [33] (Figure 16). Moreover, MSCs can directly secret anti-inflammatory cytokines (IL-4 and IL-10) and neurotrophic factors (BDNF, IGF-1, and NGF) [70] as well as fibrogenic factors (TGF-*β*) [70].

Granulocyte macrophage colony-stimulating factor (GM-CSF) recruits peripheral monocytes that are further stimulated by A*β* deposits and accelerates the clearance of A*β*, while TGF-*β* participates in several pathways that modulate amyloid metabolism, immunoregulation, and neuroprotection [74]. There are no available data explaining the capacity of GSI-953 to modulate inflammatory responses induced by A*β*25–35 injection except via the direct reduction of A*β* deposition.

Treatment of pregnant rats with BM-MSCs or GSI-953 significantly suppressed the A*β*25–35-induced upregulation of the apoptosis effector cleaved caspase-3 in the neonatal cortex, in agreement with Zhang et al. [75] who reported that A*β*25–35 stimulates proapoptotic Bax and promotes the inhibition of antiapoptotic Bcl2, resulting in elevated caspase-3 activity and neural death. Transplanted MSCs have also been shown to attenuate apoptotic cell death via inhibition of cleaved caspase-3 activity [76] via the secretion of seldin and survivin [77], proteins that interact with caspase-3 and block the downstream death cascade [78].

## 5. Conclusion

Intravenous injection of BM-MSCs or oral administration of the *γ*-secretase inhibitor GSI-953 (begacestat) mitigated cognitive impairments induced by the intracerebroventricular injection of A*β*25–35 in adult female rats. Further, BM-MSC and GSI-953 treatments during pregnancy prevented cortical maldevelopment in the offspring. These protective effects in the neonatal rat cortex were associated with the reduced expression of the APP and p-Tau proteins, inhibition of the microglial activation, reduced expression of proinflammatory cytokines (IL-1*β* and TNF-*α*), and downregulation of brain caspase-3, NF-*κ*B, and TGF-*β* (Figure 16). Furthermore, BM-MSC and GSI-953 treatments reversed the A*β*25–35-induced downregulation of the BDNF expression and upregulation of the GSK-3*β* activity. Therefore, these treatments could prevent A*β*25–35-induced neurodegeneration and cognitive impairment probably by reducing the A*β* plaque load, neurofibrillary tangle formation, and ensuing neuroinflammation. However, further studies using loss-of-function and/or gain-of-function mutation models are required to provide evidence for the implications of the tested mediators including IL-1*β*, TNF-*α*, TNFR, caspase-3, NF-*κ*B, TGF-*β*, BDNF, and GSK-3*β* in the A*β*25–35-induced neurodegenerative damage and cognitive impairment and their roles in the BM-MSC- and GSI-953-induced recovery.

## Figures and Tables

**Figure 1:**
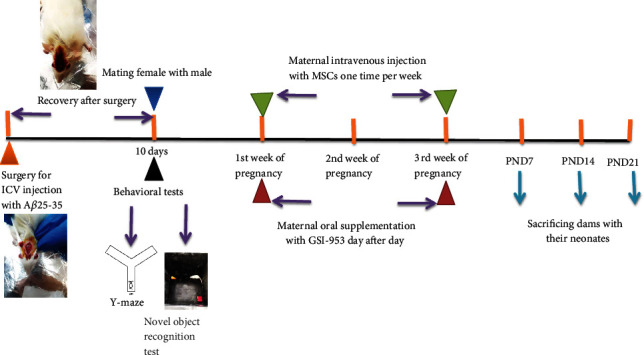
Design of the study: adult female albino rats were subjected to intracerebroventricular (i.c.v.) injection with A*β*25–35 and vehicle 0.9% saline before pregnancy and allowed to recover for 10 days and then subjected to behavioral testes (novel object recognition test and Y-maze test). After that, female rats were mated with males, and pregnant female rats subjected to A*β*25–35 injection were classified into the AD group, AD group that received intravenous injection of MSCs (one million/rat/week), and AD group orally treated with GSI-953 (2.5 mg/kg·b·wt). The neonates were sacrificed at different postnatal days (7, 14, and 21), and the brain cortex was subjected to histological, immunohistochemistry, qRT-PCR, and western blotting studies.

**Figure 2:**
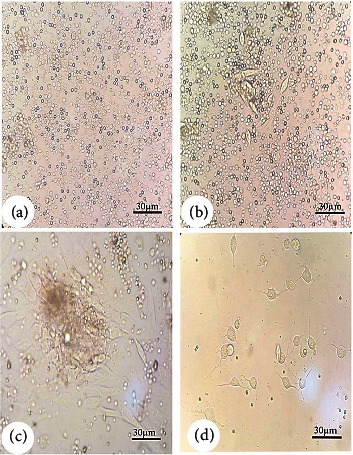
Morphology of isolated undifferentiated mesenchymal stem cells from bone marrow, under inverted microscopy. (a) MSCs in the culture flask at first day of isolation showed rounded cells, (b) MSCs begin to convert into a flat fibroblast-like morphology at 7 days of isolation, and (c, d) MSCs before injection at day 10 of isolation.

**Table 1:** Primer sequences of tested genes.

	Forward sequence	Reverse sequence
Caspase-3	TGGTTCATCCAGTCGCTTTGT	CAAATTCTGTTGCCACCTTTCG
TNFR	GGGATTCAGCTCCTGTCAAA	ATGAACTCCTTCCAGCGTGT
TGF-*β*	GTCACTGGAGTTGTACGGCA	GGGCTGATCCCGTTGATTTC
BDNF	CCGGTATCCAAAGGCCAACT	CTGCAGCCTTCCTTGGTGTA
NF-*κ*B	TTC CCT GAA GTG GAG CTA GGA	CAT GTC GAG GAA GAC ACT GGA
*β*-Actin	AGG CCC CTC TGA ACC CTA AG	GGA GCG CGT AAC CCT CAT AG

## Data Availability

This published article includes all of the data analyzed during this investigation.

## Abbreviations

AD: Alzheimer's disease
A*β*: Amyloid beta
p-Tau: Phosphorylated Tau protein
BM-MSCs: Bone marrow mesenchymal stem cells
GSI-953: Gamma secretase inhibitor-953 (begacestat)
IL: Interleukin
TNF-*α*: Tumor necrosis factor-*α*
GSK-3*β*: Glycogen synthase kinase-3*β*
BDNF: Brain-derived neurotrophic factor
NFTs: Neurofibrillary tangles
CSF: Cerebrospinal fluid
APP: Amyloid precursor protein
NGF: Nerve growth factor
DMEM: Dulbecco's modified eagle medium
BSA: Bovine serum albumin
NF-*κβ*: Nuclear factor kappa *β*
TNFR: Tumor necrosis factor receptor
TGF-*β*: Transforming growth factor *β*
PVDF: Polyvinylidene fluoride
PND: Postnatal day.
